# Postoperative adverse events after spinal surgery: Sex-specific risk profiles and ICU utilization

**DOI:** 10.1016/j.bas.2026.106018

**Published:** 2026-03-27

**Authors:** Pavlina Lenga, Meltem Ivren, Gabriel Inci, Moritz Scherer, Philip Dao Trong, Maximillian Klass, Martin Dugas, Sandro M. Krieg, Bogdana Suchorska

**Affiliations:** a Department of Neurosurgery, Heidelberg University Hospital, Heidelberg, Germany; b Medical Faculty of Heidelberg University, Heidelberg, Germany; c Department of Medical Informatics, Heidelberg University Hospital, Heidelberg, Germany; d Department of Neurosurgery, Vivantes Neukoelln, Berlin, Germany

**Keywords:** Spine surgery, Sex differences, Intensive care unit (ICU), Triage, Postoperative adverse events

## Abstract

**Background:**

Sex differences in surgical outcomes are well-documented, yet data specific to spinal surgery remain conflicting.

**Research question:**

We investigated whether postoperative risk truly differs between sexes or if disparities reflect case mix and ICU triage practices.

**Methods:**

We analyzed prospectively collected data from 1337 spinal procedures (2023–2025) at a tertiary neurosurgical center. Multivariable logistic and Poisson regression models estimated the independent effect of sex on 30-day outcomes (complications, neurological deficits, rebleeding, ICU admission) and length of stay (LOS), adjusting for age, diagnosis, procedure type, comorbidities (AACCI), and operative time.

**Results:**

The cohort included 703 men and 634 women with similar baseline diagnoses. Thirty-day adverse events were equivalent between sexes, including aggregate complications (9.8% men vs 8.8% women), new neurological deficits (2.7% vs 2.1%), and adjusted LOS (mean 8.1 days). Conversely, ICU admission was significantly more frequent in men (8.0% vs 4.1%; p = 0.005). After adjustment, female sex was not associated with complications (aOR 0.95) or LOS, but remained independently associated with reduced ICU admission (aOR 0.42, 95% CI 0.24–0.73), even when accounting for comorbidities and operative time.

**Conclusions:**

Despite equivalent complication profiles and hospital stays, women were half as likely as men to be admitted to the ICU. Since this disparity persists after adjusting for clinical complexity and operative burden, ICU utilization in spinal surgery may be driven by provider practice patterns rather than objective physiological risk.

## Highlights

• Men and women have similar 30-day complication rates and hospital stays after spinal surgery.
• Men are admitted to the ICU nearly twice as often as women after spinal surgery.
• Female sex is independently linked to lower ICU admission after adjustment for diagnosis, procedure, and comorbidities.

## 1 Introduction

**Fig. 1:**
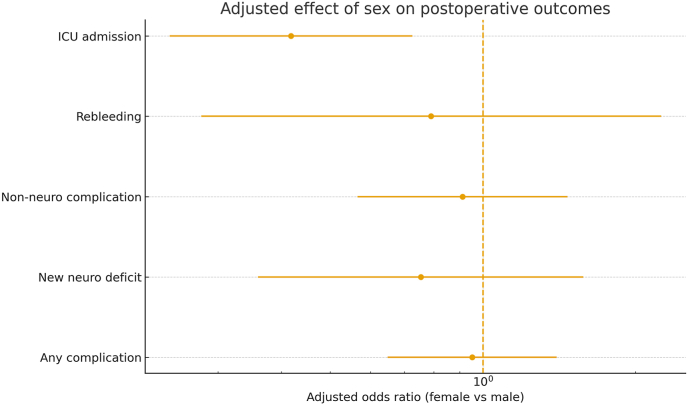
**Adjusted effect of sex on 30-day postoperative outcomes after spinal surgery.** Forest plot showing adjusted odds ratios (female vs male) with 95% confidence intervals for key postoperative endpoints derived from multivariable logistic regression models adjusted for age, primary diagnosis, and procedure class. The vertical dashed line marks an odds ratio (OR) of 1.

Sex-based disparities in surgical treatment outcomes and critical care utilization have been found across various clinical subspecialties. Especially for female patients, less treatment is provided in the ICU than for male patients in the same clinical setting (Ingram et al., 2022). In terms of acute healthcare, male patients are known to be triaged to higher levels of care or be admitted to the ICU on a timely basis for similar acute disease acuity (Ingram et al., 2022). On the contrary, women show decreased likelihood of admission concerning the ICU admissions, even if they are as or more seriously ill as the men (Todorov et al., 2021). Such sex disparity in the selection for critical care has led to concerns that ICU triage policies may not be equally sex-neutral (Todorov et al., 2021). The rationale for sex differences in the use of the ICU continues to be under investigation. Certainly there are biological and clinical implications, with men having a higher prevalence of a diagnosis of (e.g. trauma or ischemic heart disease) that necessitate much higher levels of postoperative surveillance (Weissman, 2023). Yet even accounting for such factors, evidence suggests that women receive less intensive postoperative care than men, signaling the potential for systemic bias with respect to which patients are placed on the care pathway. Allocating critical care resources proportionately to patient need, not sex, is a growing priority in perioperative medicine. In neurosurgery, especially spinal surgery, knowledge has increased on the influence of patient sex on the progression of recovery. Recent large-scale studies show that male spine surgery patients exhibit higher severe perioperative events (e.g., cardiopulmonary complications or even death) while female patients are exposed to more wound complications and longer hospital courses (Kumar et al., 2024a). These findings highlight that men's and women's risks differ following similar spine procedures. However, patients who require intensive care unit admission in spinal surgery have a great deal of difficulty or are unstable intraoperatively and still most of the patients do not use ICU level (Li et al., 2023). But there is a clear knowledge gap: Are women and men being triaged to postoperative ICUin the same ways? If women tend consistently to be less likely to gain admission to the ICU after spine intervention, the field will need to investigate whether this is simply a reflection of appropriately lower risk profiles, or whether resources were used less effectively in women in the ICU (and vice versa, whether the higher ICU use in men indicates appropriate caution, or possible overtriage). To overcome this uncertainty, we conducted the current investigation to analyze sex differences in ICU admission and 30-day postoperative outcomes for spinal surgery. We sought to establish differences in ICU utilization and short-term complication rates by patient sex by analyzing a large neurosurgical cohort. Understanding these patterns can aid our decisions for whether current ICU triage practices in spine surgery are appropriately adapted to actual patient risk or harbor some form of accidental asymmetry, thereby informing future endeavors to provide equitable and evidence-driven postoperative care (see Fig. 1).

## 2 Methods

### 2.1 Study design and ethics

This study was designed as a prospective, single-center observational cohort conducted at a tertiary care university hospital. All patients were enrolled at the hospital's neurosurgical department and observed from the day of surgery through 30 days postoperatively. The study protocol was approved by the Institutional Ethics Committee (reference number S-425/2022), and all procedures conformed to the Declaration of Helsinki and local regulations. Given the purely observational design using de-identified routine clinical data, the requirement for individual informed consent was waived by the ethics committee.

## 3 Patient population

The study population comprised all consecutive adult patients (aged **≥18 years**) who underwent any spinal surgery at the center between October 1, 2023 and August 31, 2025. Both elective (planned) surgeries and emergency spinal procedures were included, encompassing cases ranging from simple decompressions (e.g., laminectomy or discectomy) to complex instrumented fusions. Patients were excluded only if they had undergone a previous spine surgery within 30 days prior to the index procedure or if 30-day postoperative follow-up data were unavailable (lost to follow-up). This broad inclusion criterion ensured a comprehensive cohort of typical spinal surgery patients during the study period.

## 4 Perioperative management

Perioperative care was standardized across the cohort to minimize variability. All patients underwent general anesthesia administered according to uniform institutional protocols for induction, maintenance, and analgesia. Surgeries were performed by an experienced team of spine surgeons following consistent surgical techniques and postoperative care pathways. The decision for postoperative **ICU admission** was made on a case-by-case basis by the attending anesthesiologist in consultation with the surgeon, guided by intraoperative events (such as excessive blood loss or hemodynamic instability) and patient-specific risk factors. There was no fixed protocol mandating ICU admission for spinal cases; rather, admission was based on clinical judgment. Patients not requiring ICU care were observed in the post-anesthesia care unit until stable and then transferred to a standard neurosurgical ward for routine postoperative monitoring according to institutional standards. Indications for ICU admission (e.g., hemodynamic instability, airway/ventilation monitoring, or postoperative surveillance due to suspected/known OSAS) were not captured as structured variables; therefore, we could not distinguish medically necessary ICU admissions from precautionary monitoring.

Data Collection and Outcome Assessment.

All data were collected prospectively by the investigative team using a dedicated database for spine surgery outcomes (Dao et al., 2023). Baseline variables recorded for each patient included demographics (age, sex), body mass index, and comorbidities; the comorbidity burden was quantified using the age-adjusted Charlson Comorbidity Index (CCI) (Zhou et al., 2022). The primary diagnosis prompting surgery was noted and later categorized into major groups (degenerative spine disease, traumatic injury, neoplasm, infection, or other). Perioperative details such as the type of procedure (decompression-only vs. instrumented fusion, number of spinal levels involved, approach route) and surgical urgency (elective vs. emergency) were documented for each case. The study's primary outcomes were postoperative ICU admission (yes/no for each patient) and the occurrence of any 30-day adverse event (30-day AE). Adverse events were defined as any unfavorable medical or surgical outcome arising within 30 days of the index surgery, included all postoperative complications (e.g., surgical site infection, neurological deficit, cardiovascular or pulmonary complications) during the initial hospitalization or after discharge, as well as any unplanned readmission or reoperation within the 30-day postoperative period. An established institutional protocol was used to capture these events: a standardized postoperative adverse event form was completed for each patient at discharge and at follow-up, and the hospital's electronic records were cross-checked to identify any patient who returned for emergency care or readmission within 30 days. To ensure consistent and accurate classification of complications, all reported adverse events were reviewed in regular morbidity and mortality meetings. In cases of ambiguity (for instance, if it was unclear whether a medical issue was related to the surgery), the event was adjudicated by at least two senior clinicians to reach a consensus. This rigorous review process helped maintaining uniformity in determining which events qualified as complications or adverse events across the cohort. Data quality was further ensured by periodic audits of the registry against source medical records to correct any discrepancies.

### 4.1 Statistical analysis

All analyses were performed using R (version 4.3.1, R Foundation for Statistical Computing) and SPSS (version 29.0, IBM Corp., Armonk, NY, USA). Continuous variables were summarized as mean ± standard deviation (SD) or median with interquartile range (IQR), based on distribution. Categorical variables were reported as frequencies and percentages. Univariable comparisons between male and female patients used the Student's t-test or Mann–Whitney *U* test for continuous variables and chi-square or Fisher's exact test for categorical variables, as appropriate. For binary postoperative outcomes (e.g. ICU admission, any complication, new neurological deficit), we calculated: Risk differences (RD) with 95% confidence intervals (CI) using Wald approximation, Risk ratios (RR) with 95% CI, and p-values based on chi-square or Fisher's exact tests. Multivariable analyses were performed using logistic regression models with postoperative outcomes as dependent variables. Each model included sex (female vs male) as the main variable of interest and was adjusted for age (continuous), diagnosis category (degenerative, trauma, tumor, infection), and procedure type (instrumentation vs decompression). Adjusted odds ratios (aORs), 95% CIs, and p-values were reported. In a separate model, we included the age-adjusted Charlson Comorbidity Index (ACCI) to assess robustness. A sensitivity model additionally adjusted for operation time (minutes). For length of stay (LOS), we used Poisson regression with robust variance estimation to account for overdispersion. The model included the same covariates (sex, age, diagnosis, procedure), and results were presented as incidence rate ratios (IRRs) with 95% CIs. We tested for statistical interaction between sex and procedure group (sex × procedure) using a multiplicative interaction term in the logistic regression model for ICU admission. Additional sensitivity analyses incorporated baseline cardiovascular comorbidity (any) and operative time (minutes; complete-case). A two-sided p-value <0.05 was considered statistically significant in all tests.

## 5 Results

### 5.1 Patient characteristics

The study cohort comprised 1337 patients, including 703 males and 634 females. While male patients were statistically older (mean age 62.9 ± 15.3 years) than female patients (mean age 61.1 ± 16.4 years; p = 0.033), the overall distribution of clinical risk factors was highly comparable. Critically, there was no statistically significant difference between sexes in the primary diagnosis requiring surgery (degenerative, trauma, tumor, or infection; p = 0.174) or the complexity of the procedure performed (decompression vs. instrumentation; p = 0.323). Operative time was similar between sexes (median 125 [IQR 90–193] vs 123 [IQR 85–191] minutes; p = 0.381; Table 1). Median AACCI scores were statistically lower in female patients (3.0 [IQR 2.0–4.0]) than in males (3.0 [IQR 2.0–5.0]; p < 0.001, Mann–Whitney *U* test), indicating a lower comorbidity burden among women. Baseline characteristics are depicted in Table 1. Baseline cardiovascular comorbidity (any of hypertension, coronary artery disease, prior myocardial infarction, atrial fibrillation, heart failure, prior stroke, or prior venous thromboembolism) increased strongly with age and was slightly more prevalent in men overall (198 (28.2%) vs 159 (25.1%); p = 0.226). Sex differences were small within each age stratum (Supplementary Table S1).

**Table 1:** Baseline characteristics by sex.

Variable	Male	Female	p-value
Age (years) — mean ± SD	62.9 ± 15.3	61.1 ± 16.4	**0.039**
Diagnosis — Degenerative n (%)	524 (74.5%)	460 (72.6%)	0.448
Diagnosis — Trauma n (%)	71 (10.1%)	72 (11.4%)	0.513
Diagnosis — Tumor n (%)	71 (10.1%)	80 (12.6%)	0.172
Diagnosis — Infection n (%)	37 (5.3%)	22 (3.5%)	0.144
Procedure — Instrumentation n (%)	307 (44.9%)	257 (42.1%)	0.270
Procedure — Decompression n (%)	376 (55.1%)	354 (57.9%)	0.420
AACCI (median, IQR)	3.0 (2.0–5.0)	3.0 (2.0–4.0)	**< 0.001**
Operative time (min) — median (IQR)	125 (90–193)	123 (85–191)	0.381
Baseline cardiovascular comorbidity (any) — n (%)	198 (28.2%)	159 (25.1%)	0.226

Diagnosis distribution by sex (n, %), Abbreviations: SD, standard deviation; IQR, interquartile range; AACCI, age-adjusted Charlson Comorbidity Index. *Bolded p-value indicate statistically significant results.*

### 5.2 Univariate outcomes: equivalence in complications but disparity in ICU use

In the unadjusted analysis male and female patients demonstrated statistically equivalent rates across all primary complication metrics: any postoperative complication (9.8% vs. 8.8%; p = 0.590), new neurological deficit (2.7% vs. 2.1%; p = 0.549), non-neurosurgical complication (6.9% vs. 6.2%; p = 0.698), and rebleeding (1.3% vs. 0.9%; p = 0.750). The sole significant difference in resource utilization was observed in the raw rates of postoperative Intensive Care Unit (ICU) admission: 8.0% of male patients required ICU admission compared to only 4.1% of female patients (p = 0.005) (Table 2).

**Table 2:** Univariate outcome comparisons by sex.

Outcome	Male Rate	Female Rate	Risk Ratio (F/M)	RR 95% CI	p-value
Postop complication (any)	0.098	0.088	0.897	0.641–1.255	0.590
New neurological deficit	0.027	0.021	0.759	0.378–1.524	0.549
Non-neurosurgical complication	0.069	0.062	0.901	0.599–1.356	0.698
Rebleeding	0.013	0.009	0.739	0.265–2.065	0.750
**ICU admission**	**0.080**	**0.041**	**0.515**	**0.327**–**0.809**	**0.005**

Abbreviations: RR, Risk Ratio; CI, Confidence Interval, ICU, Intensive Care Unit. *Bolded p-value indicate statistically significant results.*

### 5.3 Multivariable analysis: the independent effect of sex

To isolate the independent effect of sex, multivariable regression models were applied (Table 3). After rigorous adjustment for age, diagnosis, and procedure class, female sex was definitively *not* associated with increased odds of any adverse event or prolonged hospitalization. The adjusted odds ratios (aORs) for female sex versus male were close to unity for: any postoperative complication (aOR 0.952; p = 0.803), non-neurosurgical complication (aOR 0.912; p = 0.705), and new neurological deficit (aOR 0.754; p = 0.454). Hospital length of stay (LOS) was also functionally identical, with an adjusted Incidence Rate Ratio (IRR) of 0.999 (p = 0.976). By contrast, the lower rate of ICU admission persisted as a robust, independent effect. Female sex was independently associated with reduced postoperative ICU admission (aOR 0.418; 95% CI 0.241–0.726; p = 0.002), with an adjusted absolute difference in ICU admission probability of approximately −3.2 to −3.6 percentage points.Hospital length of stay (LOS), modeled using Poisson regression with robust variance, did not differ by sex (IRR 0.999; 95% CI 0.932–1.071; *p* = 0.976; adjusted mean LOS 8.1 days in both sexes). In a sensitivity model additionally adjusting for baseline cardiovascular comorbidity, the association persisted (female vs male aOR 0.386, 95% CI 0.219–0.679), while cardiovascular comorbidity independently predicted ICU admission (aOR 3.068, 95% CI 1.695–5.551; Supplementary Table S2).

**Table 3:** Adjusted Sex Effects (Female vs. Male) on Postoperative Outcomes.

Outcome	Adjusted Odds Ratio (aOR)	95% CI	p-value
Postoperative complication (any)	0.952	0.649–1.398	0.803
New neurological deficit	0.754	0.360–1.578	0.454
Non-neurosurgical complication	0.912	0.566–1.469	0.705
Rebleeding	0.790	0.278–2.247	0.658
ICU admission	0.418	0.241–0.726	**0.002**

Abbreviations: OR, Odds Ratio; CI, Confidence Interval, ICU, Intensive Care Unit. *Bolded p-value indicate statistically significant results.*

### 5.4 The primacy of diagnosis: risk predictors outweigh sex

A sensitivity analysis of the ICU admission model demonstrated that the effect of baseline diagnosis was profoundly greater than the effect of patient sex. While female sex reduced the odds of ICU admission by 58%, high-risk diagnoses increased the odds by orders of magnitude (Table 4). Specifically: A diagnosis of infection increased the odds of ICU admission by 37-fold (aOR 36.770; p < 0.001) compared to a degenerative diagnosis. A diagnosis of trauma increased the odds of ICU admission by 12-fold (aOR 12.285; p < 0.001) compared to a degenerative diagnosis. Similarly, for non-neurosurgical complication, highly significant predictors included age (aOR 1.052 per year, p < 0.001), Infection (aOR 6.064, p < 0.001), and trauma (aOR 3.217, p < 0.001), while female sex had a non-significant aOR of 0.912 (p = 0.705).

**Table 4:** Comparative Predictive Power of Sex vs. Diagnosis (Adjusted Odds Ratios).

Outcome	Predictor	Adjusted OR (aOR)	95% CI	p-value
**ICU Admission**	Female Sex (vs. Male)	0.418	0.241–0.726	**0.002**
	Age (per year)	1.015	0.998–1.033	0.089
	Diagnosis: Infection (vs. Degenerative)	36.770	17.107–79.035	**<0.001**
	Diagnosis: Trauma (vs. Degenerative)	12.285	6.012–25.105	**<0.001**
	Diagnosis: Tumor (vs. Degenerative)	3.417	1.367–8.540	**0.009**
**Non-Neuro Complication**	Female Sex (vs. Male)	0.912	0.566–1.469	0.705
	Age (per year)	1.052	1.031–1.073	**<0.001**
	Diagnosis: Infection (vs. Degenerative)	6.064	2.888–12.731	**<0.001**
	Diagnosis: Trauma (vs. Degenerative)	3.217	1.737–5.956	**<0.001**
	Procedure: Instrumentation (vs. Decomp)	2.183	1.231–3.871	**0.008**

Abbreviations: OR, Odds Ratio; CI, Confidence Interval, ICU, Intensive Care Unit. *Bolded p-value indicate statistically significant results.*

### 5.5 Subgroup analysis of ICU admission

The independent effect of sex on ICU utilization appeared concentrated in higher-risk procedures. A procedure-stratified analysis indicated that the greatest difference in ICU admission rates occurred in patients undergoing instrumentation (adjusted OR 0.31 for females; p = 0.0006). In contrast, the difference was minimal and non-significant in the decompression-only subgroup (adjusted OR 0.82; p = 0.703). The interaction term for sex \times procedure was directional but did not reach statistical significance (p = 0.115). OSAS by age and sex is depicted by the Supplementary S2 Table. Cardiovascular comorbidity independently predicted ICU admission (aOR 3.170, 95% CI 1.742–5.768), whereas documented OSAS was not associated with ICU admission (aOR 0.391, 95% CI 0.038–4.027; Supplementary Table S4).

### 5.6 Sensitivity analyses including AACCI and operation time

To explore the role of comorbidity and operative complexity, we repeated the ICU models including AACCI and, in a further step, AACCI plus operation time. Higher comorbidity burden and longer operative time were, as expected, associated with ICU admission (Supplementary Table S3). Importantly, the protective association of female sex with ICU admission remained stable across these models; in a complete-case model additionally adjusting for baseline cardiovascular comorbidity and operative time, female sex remained associated with lower ICU admission (aOR 0.414, 95% CI 0.233–0.736; p = 0.003; Supplementary Table S3). Among patients with baseline cardiovascular comorbidity (any) (n = 357), ICU admission occurred in 31/198 men (15.7%) and 19/159 women (11.9%) (Fisher's exact p = 0.359). In an adjusted model restricted to this subgroup (age, diagnosis, procedure class), female sex remained associated with lower odds of ICU admission (aOR 0.415, 95% CI 0.189–0.914; p = 0.029). To better inform risk-based postoperative ICU triage, we additionally report the full complete-case multivariable ICU model (including diagnosis, procedure class, baseline cardiovascular comorbidity, and operative time), which identifies infectious and traumatic indications, baseline cardiovascular comorbidity, and longer operative time as the strongest independent predictors of ICU admission (Supplementary Table S5).

## 6 Discussion

In this large, adjusted spinal surgery cohort, we found no significant sex-based differences in 30-day adverse events. Female and male patients had similar rates of overall postoperative complications, new neurological deficits, non-neurosurgical events, and rebleeding. Hospital length of stay was also comparable. These findings suggest that biological sex is not an independent predictor of early postoperative morbidity when age, diagnosis, and procedure type are accounted for. Our data are consistent with previous registry-based analyses showing that, once baseline comorbidity and surgical complexity are controlled, sex alone does not confer increased risk for 30-day surgical complications in spine surgery (Kumar et al., 2024a; Dao et al., 2023). This absence of disparity held across all adverse event categories in our analysis, supporting the view that male and female patients undergoing spinal surgery experience equivalent short-term surgical safety when treated in a modern, standardized setting. These findings contrast with earlier reports of sex differences in individual complications (e.g., pulmonary in men, wound infections in women), and suggest that observed differences in previous studies may reflect differences in baseline characteristics rather than sex itself. Importantly, in our cohort women had a slightly lower AACCI than men, yet adjustment for AACCI in sensitivity analyses did not reveal any hidden sex effect on 30-day adverse events. This supports the interpretation that, once comorbidity burden is explicitly accounted for, early postoperative risk is comparable between women and men and is primarily driven by clinical variables such as diagnosis and procedure class rather than sex per se.

### 6.1 Gender disparities in ICU admission after spinal surgery

In our sub-set, female patients were much less likely than men to be admitted to the intensive care unit (ICU) after spinal surgery, and this difference persisted after adjustment for age, diagnosis category and procedure class, as well as in sensitivity analyses additionally accounting for baseline cardiovascular comorbidity and operative time (Supplementary Table S3). Crucially, the decreased intensive care unit admission in our group did not correspond to inferior short-term status: 30-day complication rates and mortality were equivalent or slightly lower (Weissman, 2023). These results suggest that reduced use of the ICU in women may not simply be due to a diminished biological need, but also underlie interpretations of gender-related disparities in postoperative triage Our information is in accordance with existing ICU literature, as women continue to be grossly underrepresented in ICU admissions, irrespective of severity of illness (Merdji et al., 2023). Women suffered significantly lower odds of admission than men of entering the ICU for similar or more advanced morbidity in a nationwide Swiss study involving >450,000 patients. Such disparities will persist in one of the highest ranked health care systems “worldwide; ” the authors confirmed, emphasizing the role of structural inequalities and unconscious bias in the triage decisions (Merdji et al., 2023). In our data, this ICU disparity also persisted after additional adjustment for comorbidity measures and operative time, indicating that measured case-mix differences do not fully explain why women are admitted to ICU less often. Taken together, these observations suggest that differences in measured case mix and comorbidity burden do not fully account for the ICU gap; however, unmeasured factors influencing ICU triage (e.g., intraoperative hemodynamic instability, blood loss, airway/ventilation considerations) were not captured as standardized variables, so residual confounding and selection bias cannot be excluded. Men in our cohort showed a higher prevalence of baseline cardiovascular comorbidity and more frequently documented OSAS. However, in sensitivity analyses additionally adjusting for cardiovascular comorbidity and reported OSAS, female sex remained independently associated with lower ICU admission, while OSAS itself was not independently associated with ICU use. Notably, the sex difference in ICU admission was not limited to patients without baseline cardiovascular disease. Within the subgroup with baseline cardiovascular comorbidity, crude ICU admission rates were numerically higher in men, and adjusted analyses suggested persistently lower ICU admission odds in women. These findings suggest that differences in comorbidity burden alone do not fully account for the observed sex disparity in postoperative ICU admission.

### 6.2 Equivalent outcomes in women challenge ICU necessity assumptions

Despite lower ICU exposure, women in our cohort recovered as well as or better than men. This mirrors findings in general surgery: a large NSQIP study (>1.4 million patients) reported lower odds of complications in women after adjustment for comorbidity, and no difference in 30-day mortality (Al-Taki et al., 2018). Weissman et al. similarly observed that men are more often admitted to intermediate or intensive care units postoperatively, yet concluded that clinical need alone does not account for the disparity (Weissman, 2023). They raised the possibility that gender bias—not risk—is influencing ICU access (Weissman, 2023). Our results reinforce this concern. Women consistently achieving equal outcomes without ICU monitoring raises the question of whether some male patients are over-triaged, consuming critical care resources that may not improve outcomes. While our elective setting limits the generalizability to acute care, our data suggest that aggressive ICU use is not universally required for a favorable recovery—particularly if care on standard wards is adequately resourced. The stability of the sex effect on ICU admission after controlling for AACCI also suggests that comorbidity-driven “true risk” is not the main driver of ICU allocation in our cohort. At the same time, the absence of worse outcomes in women should not be interpreted as evidence that ICU access is unimportant for them. Under-triage may go unrecognized in controlled cohorts, but in higher-risk populations—such as cardiac arrest or trauma—lower ICU admission rates in women have been linked to higher ICU mortality (Weissman, 2023; Merdji et al., 2023). This suggests that lower ICU use may not reflect lower clinical need, but rather potential differences in triage practices or unconscious biases in clinical decision-making.

### 6.3 Surgical field context: neurosurgery, orthopedics, and cardiac surgery

Our findings support those in neurosurgery and orthopedic spine literature, where women have consistently demonstrated non-inferior short-term outcomes. A recent meta-analysis of 124 spine surgery studies found men had higher risk of 30-day mortality and cardiopulmonary complications, while women were more prone to minor wound complications (Kumar et al., 2024b). These findings suggest that ICU avoidance in women is not associated with missed critical events. Orthopedic data reinforce this: complication profiles differ by sex, but neither group fares categorically worse (Singh et al., 2013). Only in cardiac surgery is the pattern reversed—women show higher mortality after bypass or valve procedures (Salamanna et al., 2022) (Dixon et al., 2022). However, these disparities are attributed to later referral, older age, smaller vessel size, and comorbidity burden at presentation—not intrinsic female physiology (Salamanna et al., 2022) (Dixon et al., 2022). In short, outcomes disparities are driven by care delivery patterns and systemic delay, not by sex itself.

### 6.4 Clinical implications and the path forward

Our results support a more individualized, risk-based ICU triage model. Women recovered well without routine ICU care, suggesting many spine surgery patients may not require it—regardless of sex. This aligns with efforts to reduce unnecessary ICU stay via enhanced recovery protocols. Still, the possibility of under-triage in women warrants further evaluation, particularly if unconscious bias leads clinicians to underestimate risk or symptoms. Merdji et al. emphasize that such bias—though unintentional—may shape critical care decisions (Ingram et al., 2022). The registry captured ICU admission as a binary outcome but did not provide structured data on the intensity of ICU care (e.g., ventilation duration, catecholamine requirement, or ICU-specific medication and monitoring) nor ICU length of stay. In addition, explicit indications for ICU admission (e.g., intraoperative instability, blood loss, airway risk) were not captured as standardized variables, which limits causal inference and leaves potential for residual confounding. Consequently, we cannot assess whether men were transferred to ICU with lower subsequent resource utilization (suggesting a lower admission threshold) or whether they required comparable or greater postoperative support than women. Our study reinforces that ICU triage should be guided by objective risk rather than assumptions about sex-based resilience. Incorporating structured comorbidity indices into decision-making may help standardize risk assessment across sexes; however, our sensitivity analyses show that even after additional adjustment for comorbidity measures and operative time, sex-related differences in ICU admission persist (Supplementary Table S3), implying that comorbidity scoring alone is insufficient to remove potential gender effects. Future tools may therefore benefit from sex-sensitive calibration to avoid underestimation of risk in women while also preventing unnecessary ICU admissions in men. In our complete-case multivariable model of ICU admission, the variables most strongly associated with postoperative ICU admission were an infectious indication, traumatic indication, baseline cardiovascular comorbidity and longer operative times. Although ICU admission does not necessarily equate to ICU necessity, these objective factors may serve as pragmatic, sex-neutral candidates for postoperative ICU triage and should be prospectively validated in cohorts capturing physiologic instability, blood loss, ventilation requirement, and ICU resource intensity.

## 7 Conclusion

Women in our cohort experienced fewer ICU admissions but equivalent 30-day outcomes after spinal surgery. The ICU admission difference persisted after adjustment for key measured case-mix variables, including diagnosis category, procedure class, baseline cardiovascular comorbidity and operative time. While this finding raises the possibility of sex-related differences in postoperative triage thresholds, residual confounding by unmeasured clinical factors and ICU indications cannot be excluded. However, these findings findings contribute to an emerging consensus: equity in care requires active reassessment of legacy practices, even in high-quality surgical settings.

## Consent to participate

Due to retrospective nature of the study an informed consent was waived.

## Human and animal ethics

Not applicable.

## Ethics approval

This study was conducted in accordance with the Declaration of Helsinki and approved by the local ethics committee (S 383/2024).

## Consent for publication

No individual person's data were included in this study.

## Data material availability

The datasets generated during and/or analyzed during the current study are available from the corresponding author on reasonable request.

## Authors’ contributions

All authors contributed to the study conception and design. Material preparation, data collection and analysis were performed by Bogdana Suchorska and Pavlina Lenga. The first draft of the manuscript was written by Pavlina Lenga and MS, PDT, SK, BS commented on previous versions of the manuscript. All authors read and approved the final manuscript.

## Authors' information

Pavlina Lenga, Department of Neurosurgery, Heidelberg University Hospital, Heidelberg, Germany; Meltem Ivren, Department of Neurosurgery, Heidelberg University Hospital, Heidelberg, Germany; Gabriel Inci, Department of Neurosurgery, Heidelberg University Hospital, Heidelberg, Germany; Maximilliana Klass, Institute of Medical Informatics, Heidelberg University Hospital, Heidelberg, Germany; Moritz Scherer, Department of Neurosurgery, Heidelberg University Hospital, Heidelberg, Germany; Philip Dao Trong, Department of Neurosurgery, Heidelberg University Hospital, Heidelberg, Germany; Martin Dugas, Institute of Medical Informatics, Heidelberg University Hospital, Heidelberg, Germany; Sandro M. Krieg, Department of Neurosurgery, Heidelberg University Hospital, Heidelberg, Germany; Bogadana Suchorska, Department of Neurosurgery, Heidelberg University Hospital, Heidelberg, Germany.

## Funding

There was no external funding for the presented work.

## Conflicts of interest

All authors declare that they have no conflicts of interest.
