# Supplementary material for: Postoperative adverse events after spinal surgery: Sex-specific risk profiles and ICU utilization
Source: Brain Spine. 2026 Mar 27;6:106018. doi: 10.1016/j.bas.2026.106018 (PMC13087688; doi:10.1016/j.bas.2026.106018)
Supplement: Multimedia component 1 [file mmc1.docx]

**Supplementary Table S1. Baseline cardiovascular comorbidity by sex and age group**

| Age group | Male n/N (%) | Female n/N (%) | p-value |
| --- | --- | --- | --- |
| Overall | 198 (28.2%) | 159 (25.1%) | 0.226 |
| <65 | 47 (13.5%) | 41 (11.8%) | 0.578 |
| 65–<80 | 96 (36.9%) | 71 (35.5%) | 0.828 |
| ≥80 | 55 (57.3%) | 47 (53.4%) | 0.703 |

**Supplementary Table S2. Documented OSAS by sex and age group**

| Age group | Male n/N (%) | Female n/N (%) | p-value |
| --- | --- | --- | --- |
| Overall | 18 (2.6%) | 4 (0.6%) | 0.008 |
| <65 | 6 (1.7%) | 4 (1.2%) | 0.752 |
| 65–<80 | 10 (3.8%) | 0 (0.0%) | 0.006 |
| ≥80 | 2 (2.1%) | 0 (0.0%) | 0.498 |

Supplementary Table S3. Sensitivity analyses for ICU admission (female vs male) across models

| **Model (all adjusted for age, diagnosis, procedure class)** | **N** | **Female vs Male aOR (95% CI)** | **p-value** | **Cardiovascular comorbidity aOR (95% CI)** | **Operative time aOR (95% CI)** |
| --- | --- | --- | --- | --- | --- |
| Main model | 1294 | 0.418 (0.241–0.726) | 0.002 | — | — |
| + Baseline cardiovascular comorbidity | 1294 | 0.386 (0.219–0.679) | <0.001 | 3.068 (1.695–5.551) | <0.001 |
| + Operative time (complete-case) | 1267 | 0.444 (0.253–0.779) | 0.005 | — | 1.004 (1.002–1.007) |
| + Cardiovascular comorbidity + operative time (complete-case) | 1267 | 0.414 (0.233–0.736) | 0.003 | 3.129 (1.708–5.734) | 1.004 (1.002–1.007) |

Abbreviations: aOR, adjusted odds ratio; CI, confidence interval. Operative time modeled per minute. Models restricted to cases with specified procedure class.

**Supplementary Table S4. ICU admission model including cardiovascular comorbidity and documented OSAS**

| Predictor | Adjusted OR (aOR) | 95% CI | p-value |
| --- | --- | --- | --- |
| Female sex (vs. male) | 0.377 | 0.214–0.666 | <0.001 |
| Age (per year) | 1.000 | 0.981–1.019 | 0.988 |
| Cardiovascular comorbidity (any) | 3.170 | 1.742–5.768 | <0.001 |
| Documented OSAS | 0.391 | 0.038–4.027 | 0.430 |
| Diagnosis: infection (vs. degenerative) | 29.585 | 13.530–64.694 | <0.001 |
| Diagnosis: trauma (vs. degenerative) | 11.696 | 5.605–24.406 | <0.001 |
| Diagnosis: tumor (vs. degenerative) | 3.509 | 1.394–8.831 | 0.008 |

Supplementary Table S5. Multivariable predictors of ICU admission in the complete-case model including diagnosis, procedure class, baseline cardiovascular comorbidity, and operative time

| Predictor | Adjusted OR (aOR) | 95% CI | p-value |
| --- | --- | --- | --- |
| Female sex (vs. male) | 0.414 | 0.233–0.736 | 0.003 |
| Age (per year) | 1.004 | 0.984–1.025 | 0.667 |
| Baseline cardiovascular comorbidity (any) | 3.129 | 1.708–5.734 | <0.001 |
| Operative time (per minute) | 1.004 | 1.002–1.007 | <0.001 |
| Diagnosis: trauma (vs. degenerative) | 11.555 | 5.503–24.262 | <0.001 |
| Diagnosis: tumor (vs. degenerative) | 2.959 | 1.160–7.551 | 0.023 |
| Diagnosis: infection (vs. degenerative) | 31.290 | 14.248–68.717 | <0.001 |
| Procedure: instrumentation (vs. decompression) | 1.201 | 0.590–2.445 | 0.613 |

Notes: Complete-case logistic regression model (N=1,267) restricted to cases with specified procedure class and non-missing operative time. Operative time is modeled per minute (corresponding to an aOR of 1.30 per 60 minutes).
